# Artificial intelligence with magnetic resonance imaging for prediction of pathological complete response to neoadjuvant chemoradiotherapy in rectal cancer: A systematic review and meta-analysis

**DOI:** 10.3389/fonc.2022.1026216

**Published:** 2022-10-12

**Authors:** Lu-Lu Jia, Qing-Yong Zheng, Jin-Hui Tian, Di-Liang He, Jian-Xin Zhao, Lian-Ping Zhao, Gang Huang

**Affiliations:** ^1^ The First Clinical Medical College of Gansu University of Chinese Medicine, Lanzhou, China; ^2^ Evidence-Based Nursing Center, School of Nursing, Lanzhou University, Lanzhou, China; ^3^ Evidence-Based Medicine Center, School of Basic Medical Sciences, Lanzhou University, Lanzhou, China; ^4^ Department of Radiology, Gansu Provincial Hospital, Lanzhou, China

**Keywords:** rectal neoplasms, artificial intelligence, pathological response, radiomics, magnetic resonance imaging

## Abstract

**Purpose:**

The purpose of this study was to evaluate the diagnostic accuracy of artificial intelligence (AI) models with magnetic resonance imaging(MRI) in predicting pathological complete response(pCR) to neoadjuvant chemoradiotherapy (nCRT) in patients with rectal cancer. Furthermore, assessed the methodological quality of the models.

**Methods:**

We searched PubMed, Embase, Cochrane Library, and Web of science for studies published before 21 June 2022, without any language restrictions. The Quality Assessment of Diagnostic Accuracy Studies 2 (QUADAS-2) and Radiomics Quality Score (RQS) tools were used to assess the methodological quality of the included studies. We calculated pooled sensitivity and specificity using random-effects models, I^2^ values were used to measure heterogeneity, and subgroup analyses to explore potential sources of heterogeneity.

**Results:**

We selected 21 papers for inclusion in the meta-analysis from 1562 retrieved publications, with a total of 1873 people in the validation groups. The meta-analysis showed that AI models based on MRI predicted pCR to nCRT in patients with rectal cancer: a pooled area under the curve (AUC) 0.91 (95% CI, 0.88-0.93), sensitivity of 0.82(95% CI,0.71-0.90), pooled specificity 0.86(95% CI,0.80-0.91). In the subgroup analysis, the pooled AUC of the deep learning(DL) model was 0.97, the pooled AUC of the radiomics model was 0.85; the pooled AUC of the combined model with clinical factors was 0.92, and the pooled AUC of the radiomics model alone was 0.87. The mean RQS score of the included studies was 10.95, accounting for 30.4% of the total score.

**Conclusions:**

Radiomics is a promising noninvasive method with high value in predicting pathological response to nCRT in patients with rectal cancer. DL models have higher predictive accuracy than radiomics models, and combined models incorporating clinical factors have higher diagnostic accuracy than radiomics models alone. In the future, prospective, large-scale, multicenter investigations using radiomics approaches will strengthen the diagnostic power of pCR.

**Systematic Review Registration:**

https://www.crd.york.ac.uk/prospero/, identifier CRD42021285630.

## Introduction

More than 700,000 people are diagnosed with rectal cancer each year in the world, 70% of which are locally advanced rectal cancer (LARC) (1). The current standard treatment for LARC is neoadjuvant chemoradiation followed by total mesorectal excision(TME) (2–4). However, individual responses to neoadjuvant chemoradiotherapy (nCRT) are highly heterogeneous, ranging from pathological complete responses(pCR) with no viable cancer cells to small groups of cancer cells or even a small group of patients with tumor progression. Previous studies reported that about 15-27% of patients present pCR after nCRT (5). For those patients, organ preservation methods, such as “wait-and-see” and local excision (6), can achieve a comparable survival rate with pCR as TME, decreasing TME-related morbidity and functional problems (7). However, at present, pathological complete responses can only be confirmed by histopathological examination of surgically resected specimens, so in the personalized medicine of LARC, there is an urgent need to accurately predict pCR in a timely and non-invasive manner before implementing nCRT.

In rectal cancer patients, tumor response to nCRT can be assessed by computed tomography (CT), Positron emission tomography-computed tomography (PET/CT), or rectal ultrasound. However, magnetic resonance imaging (MRI) is the most accurate method to assess and predict pCR after nCRT (8–10). MRI is the imaging modality with the highest soft-tissue contrast. Rectal MRI can accurately evaluate the tumor location, tumor stage, invasion depth, extramural vascular invasion (EMVI), and circumferential resection margin (11). Multiparametric MRI can also reflect the pathophysiological information of rectal cancer, including dynamic contrast-enhanced magnetic resonance imaging (DCE-MRI), diffusion-weighted imaging (DWI), and proton magnetic spectroscopic imaging (12–14). Changes in image morphology and image parameters extracted from contrast-enhanced MRI and DWI can help predict treatment response (15). To that end, mrTRG, a classification system similar to Mandard’s tumor regression grade (TRG) system, has been developed, based on hypointensity in T2-weighted sequences of fibrotic tissue in the lesion (16). However, the low predictive value and poor consistency of mrTRG methods for pathological TRG hinder its clinical application (17).

Artificial intelligence(AI) has been frequently and successfully applied in the field of medical image analysis and can automatically identify complex patterns in imaging. Machine learning(ML) is a branch of AI that has been widely used in rectal cancer, including radiomics and deep learning(DL). Radiomics can transform clinical images into mineable data for quantitative analysis through high-throughput extraction (18). Thus, providing non-visual information related to tumor heterogeneity and underlying pathophysiology. Combining AI algorithms and MRI is a promising tool for improving the prediction of diagnosis or prognosis in patients with rectal cancer. In rectal cancer patients, radiomics has been widely used in rectal cancer staging classification (19), rectal cancer liver metastasis (20), distant metastasis (21), colorectal cancer KRAS gene status (22), MSS status (23), aquaporin-1 expression (24) and predicting the early stage of neoadjuvant chemoradiotherapy progress (25).In recent years, several studies based on radiomics have emerged to predict the pathological response to nCRT in patients with rectal cancer, including traditional machine learning models, deep learning models, and delta models. However, no comprehensive review of current research on artificial intelligence (AI) models for predicting pathological responses to nCRT in rectal cancer patients has been conducted, and the overall effectiveness of this prediction model is unknown. Furthermore, because radiomics research is a complicated process with several phases, it is critical to evaluate the method’s quality to assure reliable and repeatable models before putting it into clinical applications.

The purpose of this systematic review was to describe available research on radiomics predicting pathological response to nCRT, evaluate the overall effectiveness of prediction models, and evaluate the methodological quality and bias risk in radiomics workflows.

## Methods

The Standards for the Reporting of Diagnostic Accuracy Studies (STARD) (26) and Preferred Reporting Items for Systematic Reviews and Meta-Analyses (PRISMA) (27) guidelines were followed. CRD 42021285630 is the registration number.

### Search strategy

We searched from the databases of PubMed, Embase, Cochrane Library, and Web of science, for studies conducted before June 20, 2022. Using the technique of blending topic and free words. The key topic terms were “Rectal Neoplasms”, “Artificial Intelligence”, and “Magnetic Resonance Imaging”, as well as related terms. The search strategy and detailed procedures are demonstrated in **Table S1**.

### Inclusion and exclusion criteria

Studies that matched the following criteria were chosen after duplicate literature was eliminated (1): Pathologically proven locally advanced rectal cancer patients (T3/T4 and/or N1+) (2); All patients received neoadjuvant chemoradiation treatment(traditional long course and trial regimens were included) (3); Use of MRI as the examination modality (if other imaging modalities are used, as long as MRI has been studied separately) (4); Predicting pathological responses in patients using artificial intelligence models (5); Provided the information necessary for the reconstruction of 2 × 2 contingency tables (6); Any study design, including retrospective and prospective observational studies (7); the language of the publication was English.

The following criteria were used to exclude our studies (1): each study had at least 10 patients (2); Case reports, review articles, letters, meeting reports, and editorials (3); Studies that included neoadjuvant chemotherapy only (4); Studies that included neoadjuvant radiotherapy only (5); Classification of patients as responsive and non-responsive, rather than pathological complete and non-pathological complete responses (6); No validated studies. The titles and abstracts of all identified studies were examined first, followed by a full-text review of possibly suitable articles.

### Data extraction

The following information was extracted from the eligible articles (1): study characteristics: authors (years of publication), country of corresponding author, study type, and study design (2); participants characteristics: neoadjuvant chemoradiotherapy, operation, standard reference, image examination interval, MRI scan parameters (3); model characteristics: image, region of interest (ROI) segmentation, input data, feature selection, modeling methods, verification methods (4); AI model performance: AUC, sensitivity, specificity, pCR population and non-pCR population.

### Assessment of study quality

The Quality Assessment of Diagnostic Accuracy Studies 2 (QUADAS-2) and Radiomics Quality Score (RQS) were used to evaluate the included studies’ methodological quality and study-level risk of bias, respectively. The RQS was proposed by Lambin (28) in 2017 to evaluate radiomics research based on five stages of radiomics research (data selection, medical imaging, feature extraction, exploratory analysis, and modeling). The RQS tool has a total of 16 key items for quantifying the radiomics workflow. Details are in **Table S2**. The QUADAS-2 standard consists of four parts: patient selection, index test, reference standard, and flow and timing (29), which are detailed in **Table S3**. To obtain a consensus, two graduate students separately rated the quality and discussed disputes with the evidence-based medicine teacher.

### Statistical analysis

We analyzed the raw data with the Midas command in Stata software (30), and we estimated the pooled sensitivity, specificity, positive likelihood ratio (PLR), negative likelihood ratio (NLR), and diagnostic odds ratio (DOR) with 95% CI using a bivariate random-effects model. We created a receiver operating characteristic curve (ROC) with sensitivity on the X-axis and specificity on the Y-axis, as well as the area under the curve (AUC) to demonstrate the diagnostic power of the included research (31).

We used linked forest plots to compare research and discover heterogeneity in confluent sensitivity and specificity (32). We initially visually inspected ROC images and forest plots to examine heterogeneity between study results. The I^2^ measure was used to assess heterogeneity in studies. I^2^ values >75% are highly heterogeneous (32). Two-sided P<0.05 was considered statistically significant. We plan to perform subgroup analyses to investigate potential sources of heterogeneity. As possible sources of heterogeneity, we considered modeling methods (radiomics and deep learning), sample size (whether greater than 100), radiomics feature extraction software (PyRadiomics and Others), regions of interest (2D and 3D), validation methods (external validation and internal validation) and inclusion of clinical factors (combined models and separate imaging feature models) were performed in subgroup analyses, which also allowed us to assess the impact of various factors on the model’s diagnostic performance.

We used a funnel plot visual asymmetry evaluation to identify publication bias (33), which we first published using measurements of effect magnitude plotted against measures of study accuracy. We then officially analyzed test accuracy using Deeks’ test and diagnostic odds ratio (DOR).

## Results

### Literature search

**Figure 1:**
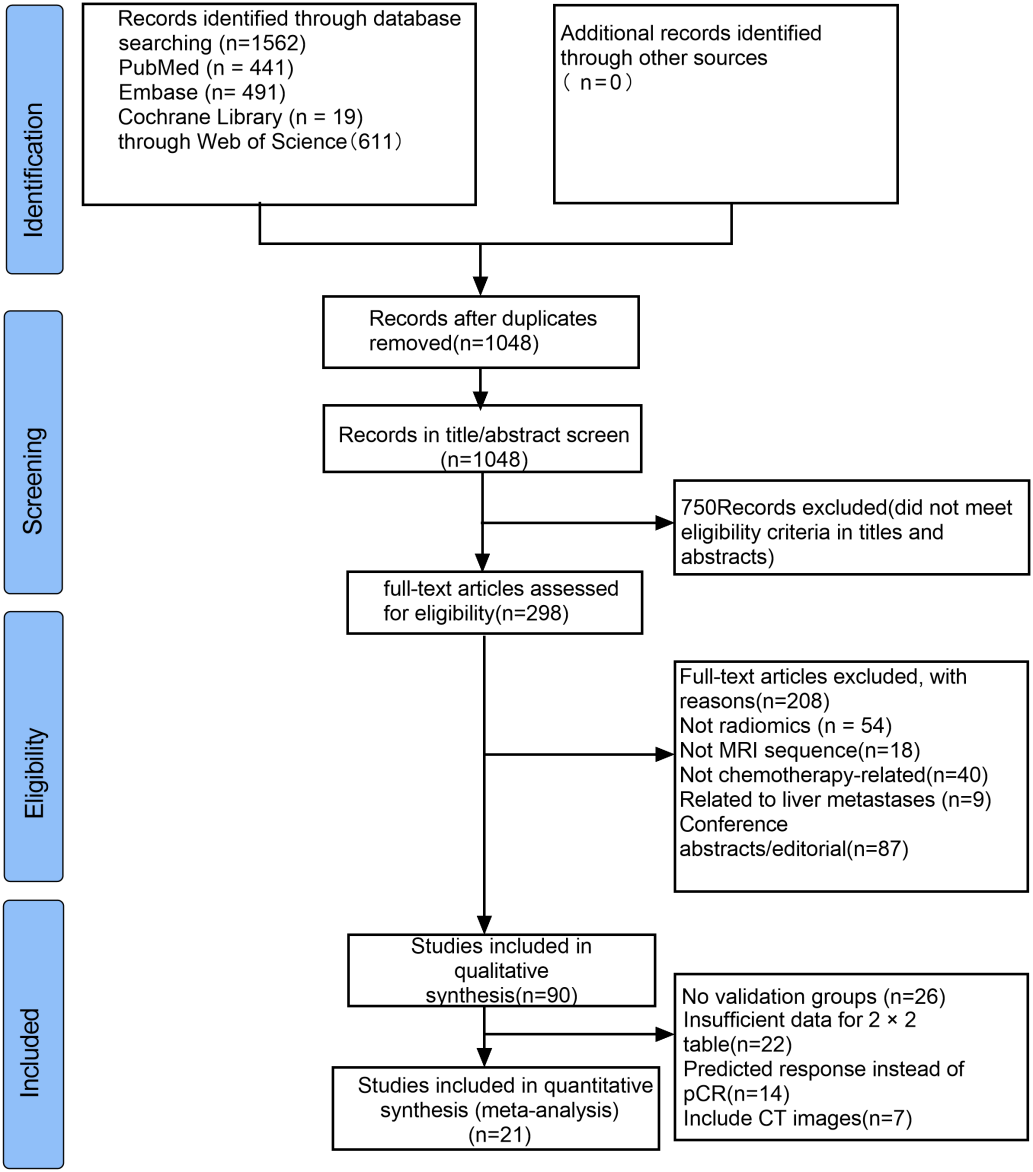
Flow diagram of the study selection process for this meta-analysis.

Through searches of PubMed, Embase, Cochrane Library, and Web of Science databases, a total of 1562 articles were retrieved. We browsed the titles and abstracts of 1048 studie, reviewed the full text of 298 studies, and finally reported from 90 articles. The application of AI models in neoadjuvant chemoradiotherapy for rectal cancer was evaluated, and finally, 21 articles were eligible for meta-analysis. The selection process is shown in **Figure 1**.

### Characteristics of included studies

The 21 included studies were published between 2018 and 2022. More than half of the studies (11/21) were based on a population from China (34–44), three from South Korea (45–47), three from Italy (48–50), two from the USA (51, 52), one from Brazil (53), and one from Belgium (54). Two studies were prospective, and all the remaining studies (19/21) had a retrospective design. These 21 studies included a total of 6913 patients with sample sizes ranging from 95 to 1033 (median: 186). The definition of pCR was the same among most of the included studies(17/21), four studies not describing the definition of pCR. Long-course radiotherapy dosesrangeg from 41.8-50.6 Gy with different concurrent chemotherapy (**Table 2**).

Eleven studies used both 1.5T and 3.0T MRI scan types, seven studies only used 3.0T MRI scan, and two studies used 1.5T MRI scan. Most studies (15/29) used two or more sequences to build their predictive models. Five studies used only T2WI sequences to construct the models, and the remaining one used DWI sequences (44). All studies included image slice thicknesses between 2.00mm and 8.0mm.

The most used segmentation software is ITK.SNAP (7/21), followed by 3D Slicer (3/21). Most studies performed manual segmentation (15/21), two studies performed semi-automatic segmentation, and one study performed automatic segmentation (39), The segmentation method was not described in the remaining three studies. Ten studies used two-dimensional(2D) segmentation, nine studies used three-dimensional(3D) segmentation, and the other two studies used an unknown segmentation approach.

The most commonly used image feature extraction software is PyRadiomics (6/21), followed by MATLAB (3/21). The number of radiomic features extracted from the images varied from 34 to 8524. To avoid possible overfitting when developing radiomic models, feature selection and dimensionality reduction must be performed because radiomic features often exceed sample size. Each study used a different approach to feature selection and dimensionality reduction, and some studies performed more than one-dimensionality reduction approach. The most commonly used are Pearson correlation and Least Absolute Shrinkage and Selection Operator (LASSO) regression. Repeatability evaluation of imaging features can also be used for feature selection. The thresholds for robust features were set at 0.6-0.915 in seven studies that performed inter-class correlation coefficient (ICC) analysis. Extracted features were described in 12 studies, of which texture features were found in 11 studies, and the features extracted in 9 studies were unknown.

Five studies used deep learning(DL) methods to build models, and the remaining sixteen studies used ML methods to build models. The most common ML classifier is logistic regression. Nine studies used external validation, eleven studies used randomization validation, and the remaining one used cross-validation (53). Fifteen studies used radiomics features alone to construct models, and six studies constructed comprehensive models that combined clinical factors and radiomics features.

The study characteristics and results are summarized in **Table 1** and **Table 2**.

**Table 1:** Summary of general study characteristics.

Study	Country	Study type	No. of patients	MRI field intensity	Sequences	Slice thickness	Image acquisition time	Radiotherapy Dose (Cumulative)	Chemotherapy Regimen	Stand Reference	Definition of pCR	AUC
Antunes 2021	USA	Retrospective	104	1.5 T, 3.0T	T2WI	3.0-8.00mm	Before nCRT	45-50.4 Gy	Capecitabine (825 to 850 mg/m^2^/ day)	Surgical pathology	0% viable tumor cells remaining after nCRT	0.712
Boldrini 2022	China	Retrospective	220	1.5 T, 3.0T	T2WI	Not reported	Before nCRT	45 Gy	Oral capecitabine 1650 mg/m2 * die (d1-7, q7); 5-fluorouracil 225 mg/m2 * die (d1-7, q7) or CapOx 60 mg/m2 of iv oxaliplatin (d1, q7) plus oral capecitabine 1300 mg/m2 * die (d1-7, q7)	Surgical pathology	Absence of tumour disease on surgical specimen	0.75
Bulens 2021	Belgium	Retrospective	125	3.0T	T2WI, DWI	3.0-5.0mm	Before nCRT	45 Gy-50 Gy	Infusion of 5-Fluorouracil 225 mg/m^2^/d), capecitabin 825 mg/m^2^ bid	Surgical pathology	ypT01N0	0.86
Cheng 2021	China	Retrospective	193	3.0T	T1W,T2WI,T2FS	3.0mm,4.0mm	Before nCRT	45.0-50.4 Gy	mFOLFOX6, CapeOX	Surgical pathology	No tumor regression	0.912
Cui 2019	China	Retrospective	186	3.0T	T2WI, CE-T1WI,ADC	3.0mm,5.0mm	Before nCRT	50 Gy	Capecitabine(800mg/m^2^/ day)	Surgical pathology	No viable tumour cells	0.966
Feng 2022	China	Prospective	1033	1.5 T, 3.0T	T2WI, CE-T1WI, DWI	2.0-6.0mm	Before nCRT	50 Gy/45Gy	5-fluorouracil based regimen combined with or without oxaliplatin	Surgical pathology	No remaining viable cancer cells	0·812
Horvat 2018	Brazil	Retrospective	114	1.5 T, 3.0T	T2WI, DWI	3.0mm	Before nCRT	Not reported	Not reported	Surgical pathology	ypT0N0	0.93
Horvat 2022	USA	Retrospective	164	1.5 T, 3.0T	T2WI, DWI	3.0mm,5.0mm	Before nCRT	Not reported	Not reported	Surgical pathology	Not reported	0.83
Jang 2021	Korea	Retrospective	466	1.5 T, 3.0T	T1W,T2WI	3.0mm	Before nCRT	50.4 Gy	Concurrent fluoropyrimidine	Surgical pathology	Not reported	0.76
Jin 2021	China	Retrospective	622	1.5 T, 3.0T	T1WI, CE-T1WI, T2WI, DWI	3.0mm,5.0mm	Before and after nCRT	Not reported	Not reported	Surgical pathology	No viable tumor cells remaining	0.97
Lee 2021	Korea	Retrospective	912	1.5 T, 3.0T	T2WI, CE-T1WI, DWI	3.0mm	Before nCRT	Not reported	Not reported	Surgical pathology	Not reported	0.837
Nardone 2022	Italy	Retrospective	100	1.5 T	T2WI, ADC, DWI	Not reported	Before nCRT	45 Gy	Capecitabine(825 mg/m^2^/ day)	Surgical pathology	No viable cancer cells	0.92
Pang 2021	China	Retrospective	275	1.5 T	T2WI	5.0mm	Before nCRT	45 Gy	Oral or intravenous 5-fluorouracil	Surgical pathology	No surviving tumor cells	0.815
Rengo 2022	Italy	Retrospective	95	1.5 T, 3.0T	T2WI	3.0mm,4.0mm	Before nCRT	45 Gy	Oxaliplatin (2-hour infusion 50 mg/m2), 5-FU 200 mg/m2/die, desamethasone (8 mg) and ondansetron (8 mg)	Surgical pathology	No viable cancer cells	0.833
Shaish 2020	Italy	Retrospective	132	Not reported	T2WI	3.0-8.0mm	Before nCRT	5 Gy	Capecitabine, 5- flourouracil, FOLFOX	Surgical pathology	Not reported	0.80
Shin 2022	Korea	Retrospective	898	1.5 T, 3.0T	T2WI, ADC, DWI	Not reported	Before and after nCRT	Not reported	Not reported	Surgical pathology	No surviving tumor cells	0.82
Wan 2019	China	Retrospective	120	3.0T	T1W,T2WI	3.0mm-5.0mm	Before nCRT	50 Gy	Capecitabine(1650 mg/m2)	Surgical pathology	Absence of residual tumor cells	0.84
Wan 2020	China	Retrospective	165	3.0T	T2WI,T1WI,DWI	3.0mm-5.0mm	Before nCRT	45-50.4 Gy	Capecitabine(825 mg/m^2^/ day), oxaliplatin(130 mg/m2)	Surgical pathology	Absence of viable tumor cells	0.91
Yi 2019	China	Retrospective	134	1.5 T, 3.0T	T1WI, CE-T1WI, T2WI	Not reported	Before nCRT	46-50Gy	Capecitabine(825 mg/m2/ day)	Surgical pathology	No viable tumor cells present	0.908
Zhang 2020	China	Prospective	383	3.0T	T2WI,T1WI,DKI	Not reported	Before and after nCRT	Not reported	Capecitabine(825 mg/m2/ day)	Surgical pathology	No viable tumor cells present	0.99
Zhu 2022	China	Retrospective	472	3.0T	DWI	4.0mm	Before nCRT	41.8-50.6 Gy	Capecitabine(825 mg/m2/ day)	Surgical pathology	Absence of any residual cancer cells	0.924

ADC, apparent diffusion coefficient; AUC, Area Under Curve; CE-T1WI, contrastenhanced T1 weighted imaging; DKI, diffusional kurtosis imaging; DWI, Diffusion weighted imaging; nCRT, neoadjuvant chemoradiotherapy; mFOLFOX6, oxaliplatin + fluorouracil; pCR, pathological complete response; T1WI, T1-weighted imaging; T2WI, T2 Weighted Image; T2FS, T2-weighted fat-suppression; USA, United States of America.

**Table 2:** Summary of artificial intelligence-based prediction model characteristics described in included studies.

Study	VOI software	Segmentation	ROI	Feature extraction software	Imaging features	No. of extraced feature	ICC evaluation(threshold)	Algorithm architecture	Validation
Antunes 2021	3D Slicer	Manual	2D	MATLAB	Textural features	764	No	RF	External validation
Boldrini 2022	Not reported	Manual	2D	MODDICOM	Skewness, Entropy	Not reported	No	Linear regression logistic	External validation
Bulens 2021	Not reported	Manual	2D	Not reported	Not reported	8524	No	LASSO regression	External validation
Cheng 2021	ITK.SNAP	Manual	3D	Pyradiomics	First-order, shape-based, texture features	5901	Yes (0.8)	Logistic regression	Split sample
Cui 2019	ITK.SNAP	Manual	2D	AK	Histogram parameters, texture features, factor features	1188	Yes(0.701 -0.915)	LASSO regression	Split sample
Feng 2022	ITK.SNAP	Manual	2D	Pyradiomics	Texture Features, First-order Features, Wavelets Features	2106	Yes (0.6)	SVM	External validation
Horvat 2018	ITK.SNAP	Manual	2D	MATLAB	Texture Features	34	No	RF	FivefoldCV
Horvat 2022	ITK.SNAP	Manual	3D	Not reported	Texture features, Haralick textures, Gabor edges	91	Yes (0.75)	RF	External validation
Jang 2021	Not applicable	Not applicable	3D	MATLAB	Not applicable	Not applicable	Not applicable	DL	Split sample
Jin 2021	Not reported	Not applicable	3D	Not reported	Not applicable	Not applicable	Not applicable	3D RP-Net	External validation
Lee 2021	3D Slicer	Semi-automatic	3D	Pyradiomics	Features on the tumor shape,voxel intensity histogram, texture of tumor areas	3740	No	LR, xgboost, lightgbm,RF, MLP, Ensemble	Split sample
Nardone 2022	Not reported	Manual	3D	LifeX	Texture Features	Not reported	Yes	Logistic regression	External validation
Pang 2021	U-Net	Automatic	2D	PyRadiomics	Not applicable	Not applicable	No	TsraU-Net	External validation
Rengo 2022	Not reported	Not reported	Not reported	WEKA	Not reported	Not reported	No	SVM,RF,J48, Naive bayes,KNN	External validation
Shaish 2020	3D slicer	Manual	3D	Pyradiomics	First-order statistics, 3D shape-based, gray-level cooccurrence matrix, gray-level run length matrix, gray-level size zone matrix, neighboring gray-tone difference matrix, gray-level dependence matrix	3190	No	Logistic regression	Split sample
Shin 2022	3D Slicer	Semi-automatic	3D	PyRadiomics	Not reported	1132	Yes (0.75)	Not reported	Split sample
Wan 2019	Not applicable	Manual	Not applicable	Not applicable	Not applicable	Not applicable	Yes (0.8)	Lasso logistic regression	Split sample
Wan 2020	Radcloud	Manual	2D	Radcloud	Shape characteristic, first-order statistical characteristics, texture features, high-order statistical characteristics	1049	No	Logistic regression	Split sample
Yi 2019	MaZda	Manual	2D	MaZda	Texture features	340	Yes (0.75)	RF, SVM	Split sample
Zhang 2020	ITK.SNAP	Manual	2D	Python	Not applicable	Not applicable	No	DL	Split sample
Zhu 2022	ITK.SNAP	Manual	3D	Python	Not applicable	Not applicable	No	CNN	Split sample

CNN, Convolutional Neural Networks; CV, cross validation; DL, Deep learning; ICC, Intra-/inter-class Correlation Coefficient; KNN, K-Nearest Neighbor; LASSO, Least Absolute Shrinkage and Selection Operator; LR, Logistic regression; MLP, multi-layer perceptron; ROI, Region Of Interes; RF, Random Forest; SVM, Support Vector Machine; 2D, Two-Dimensional; 3D, Three-Dimensional; VOI, Volume of Interest.

### RQS and risk of bias assessment

**Figure 2:**
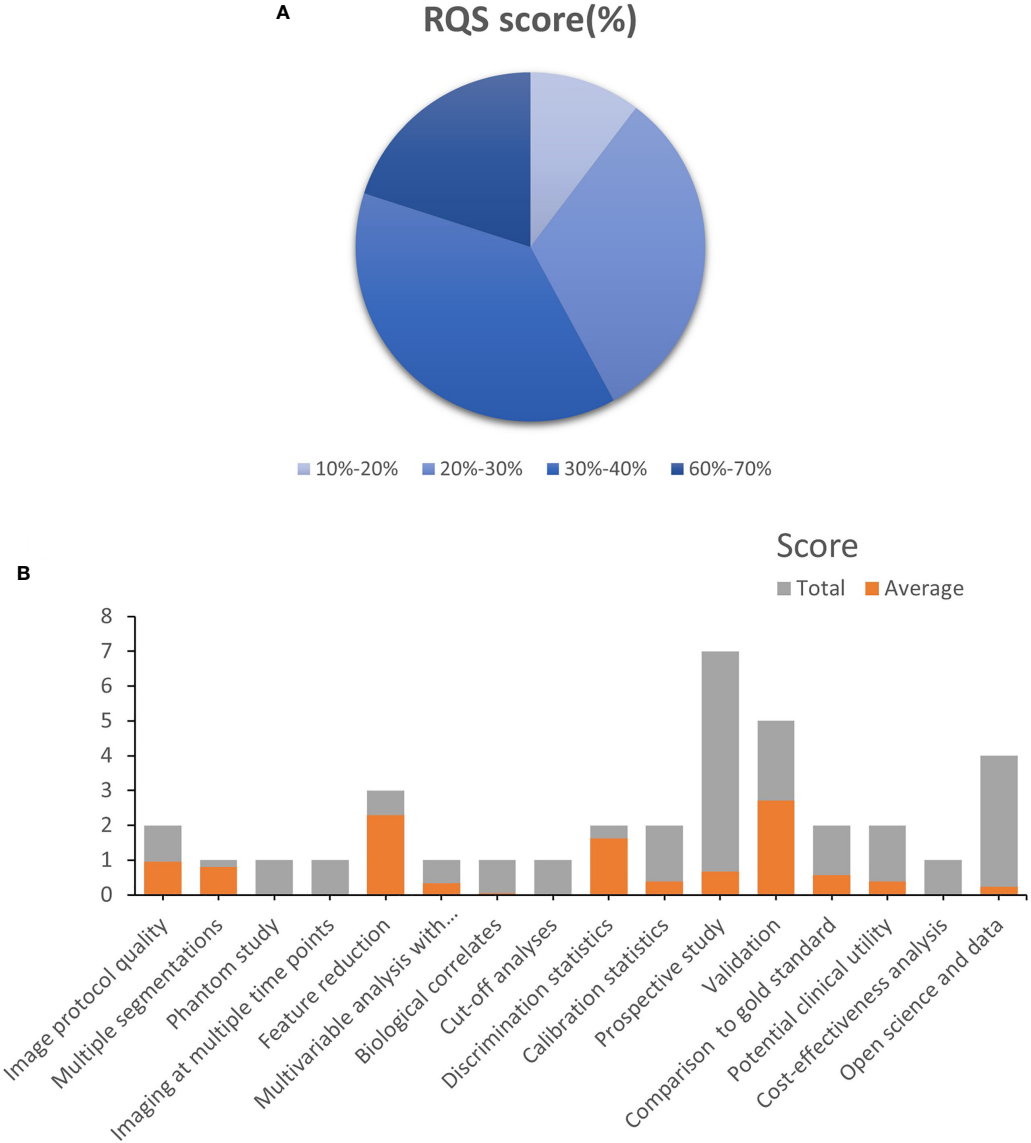
Methodological quality was evaluated by using the Radiomics Quality Score (RQS) tool. **(A)**. The proportion of studies with a different RQS percentage scores. **(B)**. Average scores of each RQS item (gray bars stand for the full points of each item, and red bars show actual points).

The included studies’ mean RQS score was 10.95, accounting for 30.4% of the overall score. Only one research (37) found the maximum RQS score of 24 (67%). Approximately half of the studies received a score of 10 or above. Because no study took into account the four elements “Phantom study on all scanners”, “Imaging at multiple time points”, “Cut-off analyses”, and “Cost-effectiveness analysis”, they obtained a score of zero. Other factors with poor average scores were “biological correlations,” “Prospective study”, “Potential clinical utility”, and “Open science and data” (**Figure 2**). A detailed description of the RQS scores is provided in **Table S4**.

**Figure 3:**
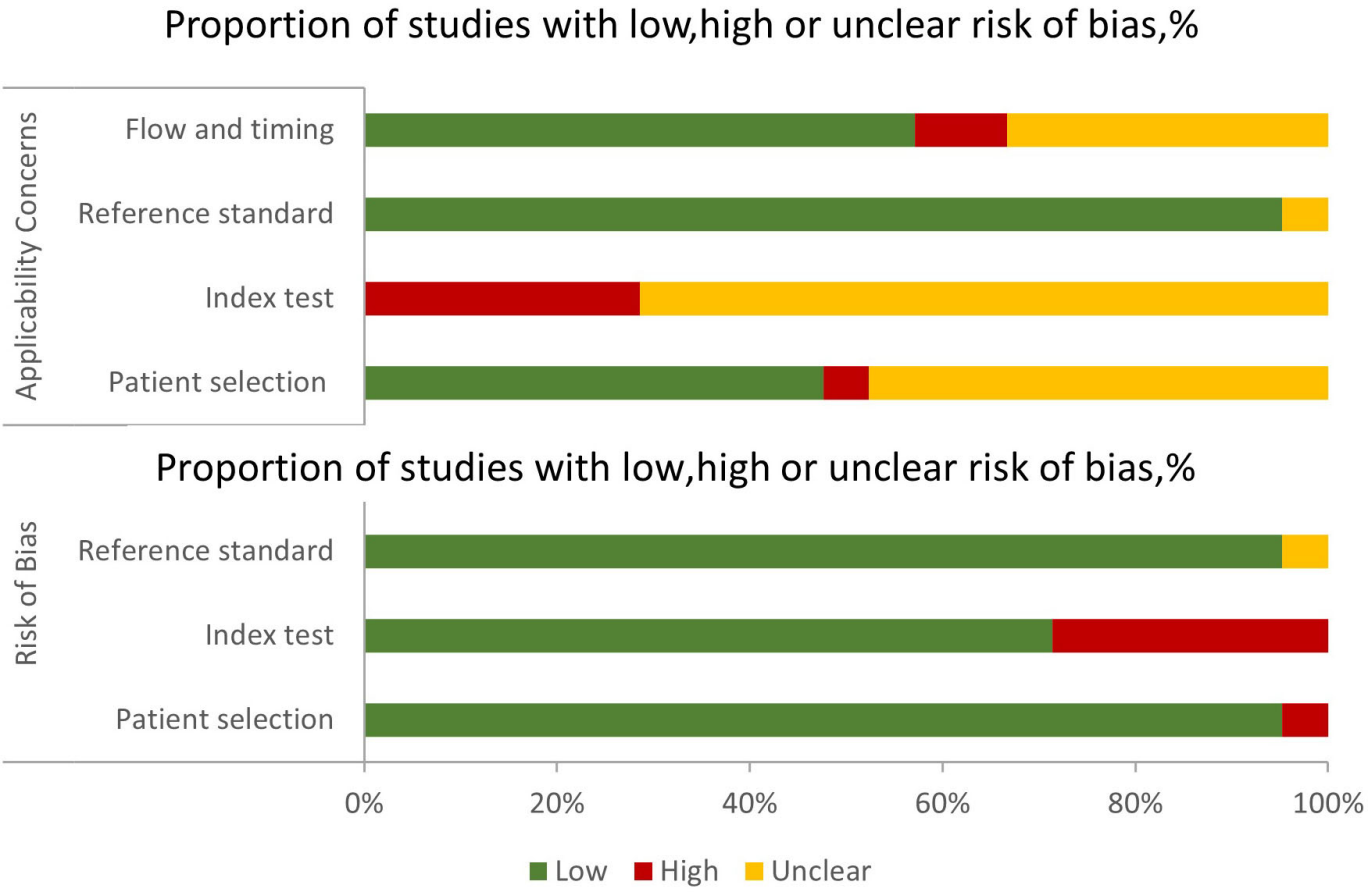
Grouped bar charts of the risk of bias and applicability concerns of the included studies were assessed by using a revised tool for the Quality Assessment of Diagnostic Accuracy Studies (QUADAS-2).

**Figure 3** depicts the risk of bias and applicability concerns for twenty-one diagnostic-related studies using QUADAS-2. In each category, the majority of research revealed a low or uncertain risk of bias (**Figure S1**). In terms of patient selection, eleven studies were deemed to have an uncertain or high risk of bias due to ambiguous methods of participant selection and/or ambiguous detailed exclusion criteria. Concerning the index test, all studies were deemed to have a high or uncertain risk of bias since it was unclear if a threshold was employed or the threshold was not pre-specified. Only one research was deemed to have an unknown risk of bias due to the lack of a description of the reference standard. Concerning the time course, nine studies were deemed to be at high or unclear risk of bias, owing to unclear gaps between indicator tests and reference standards and/or the inability to determine if all subjects got the same reference standards (**Supplemental Table S5**).

### Meta-analysis

A total of 21 studies were included in the meta-analysis, we only evaluated the validation cohorts of those studies, and radiomics assessed the efficacy of pCR after neoadjuvant chemoradiotherapy in rectal cancer: the pooled sensitivity 0.82(95% CI,0.71-0.90), pooled specificity 0.86(95% CI,0.80- 0.91), pooled PLR 6.0 (95% CI,4.0-8.9), pooled NLR 0.21(95% CI,0.12-0.35)and DOR 29(95% CI,14-61) respectively, and the pooled AUC was 0.91 (95% CI,0.88-0.93).

**Figure 4:**
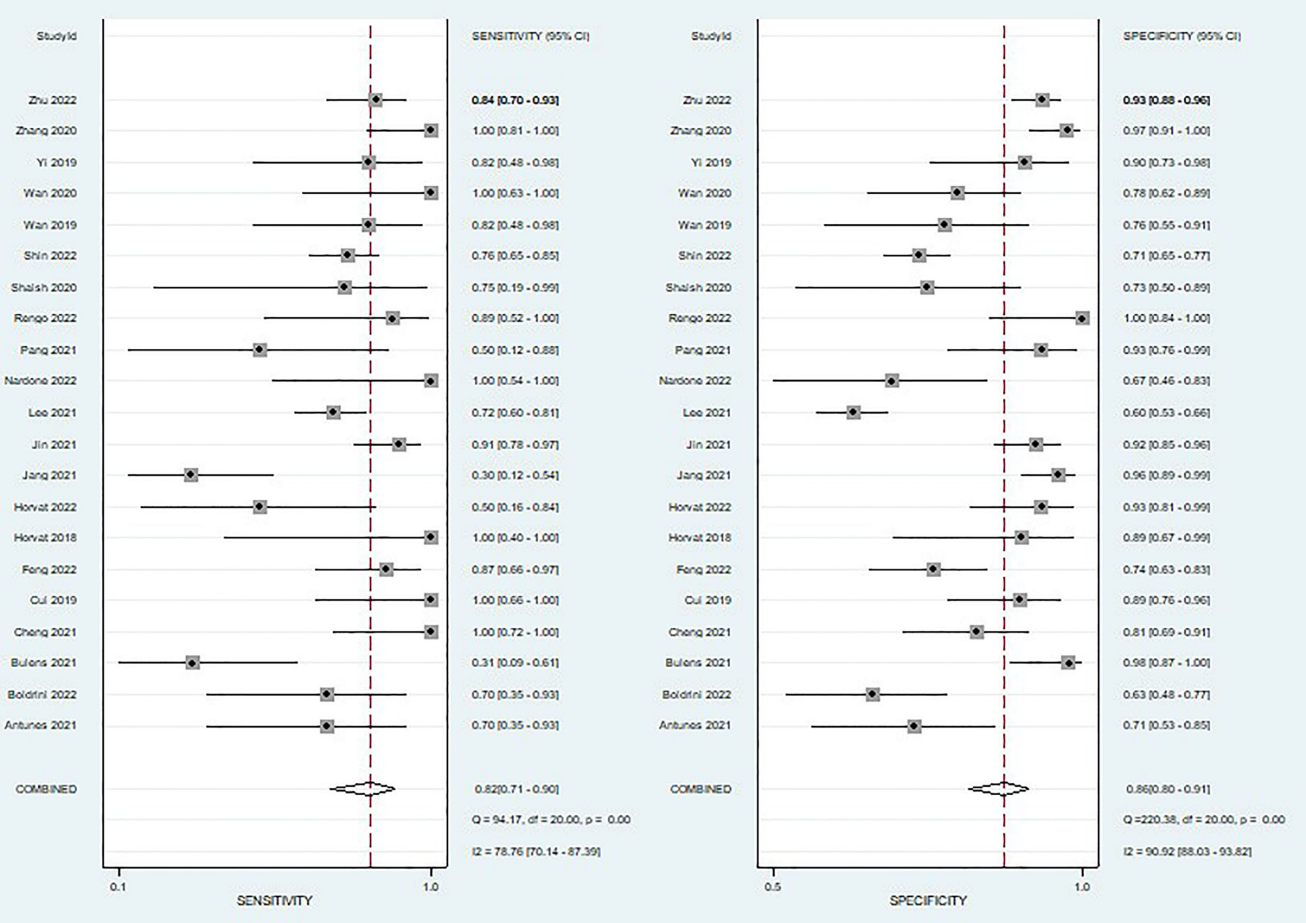
Coupled forest plots of pooled sensitivity and specificity of diagnostic performance of predicting pathological complete response to neoadjuvant chemoradiotherapy in rectal cancer. The numbers are pooled estimates with 95% CIs in parentheses; horizontal lines indicate 95% CIs.

**Figure 5:**
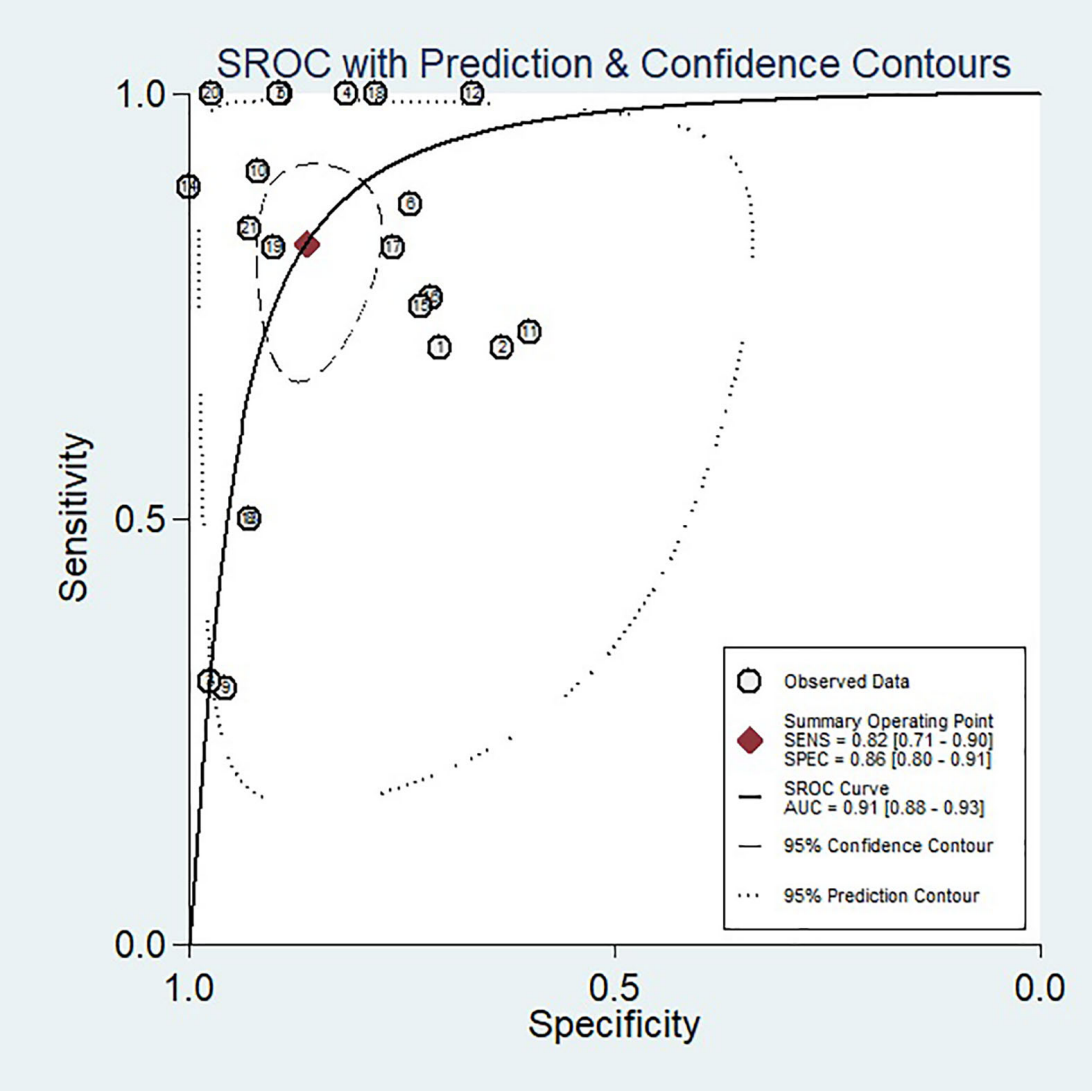
SROC curve of the diagnostic performance of artificial intelligence for the prediction of pathological complete response to neoadjuvant chemoradiotherapy in rectal cancer patients. An obvious difference was detected between the 95% CI and 95% prediction regions, indicating a high possibility of heterogeneity across the studies.

When we calculated pooled estimates, we discovered significant heterogeneity between studies in terms of sensitivity (I^2^ = 78.76%) and specificity (I^2^ = 90.92%). **Figure 4** shows the forest plot, and **Figure 5** shows the noticeable discrepancy between the 95% confidence and 95% prediction areas from the SROC curve, showing a significant probability of variability between studies.

### Subgroup analysis

To explore potential sources of study heterogeneity, we performed a subgroup analysis of 21 studies, including six different conditions and twelve subgroups. Radiomics models vary in modeling methods (radiomics and DL), sample size (whether greater than 100), radiomics feature extraction software (PyRadiomics and Others), regions of interest (2D and 3D), and validation methods (external validation and internal validation) and the inclusion of clinical factors (combined models and separate imaging feature models) showed moderate to high diagnostic value in various subgroups. The results are shown in **Table 3**.

**Table 3:** The results of subgroup analysis.

Subgroup	Number of study	Sensitivity (95% CI)	I^2^ (%)	Specificity	I^2^ (%)	PLR	I^2^ (%)	NLR	I^2^ (%)	AUC
**Modeling methods**										
Radiomic algorithm	16	0.77(0.71,0.81)	61.9	0.74(0.71,0.76)	82.1	3.47(2.66,4.54)	68.5	0.34(0.24,0.48)	54.3	0.8538
Deep learning	5	0.79(0.71,0.85)	89.5	0.94(0.91,0.96)	0.0	11.66(7.98,17.02)	0.0	0.22(0.06,0.86)	94.1	0.9724
**Sample size**										
<100	15	0.80(0.72,0.86)	70.6	0.85(0.81,0.87)	77.6	4.82(3.30,7.05)	64.2	0.27(0.15,0.49)	70.7	0.9009
>100	6	0.76(0.71,0.81)	82.9	0.77(0.74,0.80)	95.4	4.59(2.54,8.30)	92.6	0.29(0.16,0.54)	89.1	0.8771
**Radiomic software**										
PyRadiomics	6	0.76(0.70,0.82)	51.9	0.69(0.66,0.73)	79.6	2.92(2.07,4.12)	74.4	0.37(0.26,0.53)	30.3	0.8146
others	11	0.80(0.73,0.86)	78.0	0.88(0.85,0.90)	84.3	5.91(3.40,10.27)	80.4	0.20(0.08,0.48)	84.5	0.9227
**Segmentation**										
2D	10	0.79(0.71,0.87)	75.5	0.84(0.80,0.87)	81.7	5.11(3.16,8.28)	71.1	0.26(0.12,0.56)	78.4	0.9030
3D	9	0.76(0.70,0.81)	79.7	0.78(0.75,0.80)	93.2	4.45(2.72,7.26)	89.1	0.31(0.18,0.54)	83.6	0.8829
**Validation**										
External validation	9	0.77(0.68,0.84)	72.2	0.83(0.79,0.86)	84.6	4.36(2.55,7.47)	74.5	0.33(0.18,0.62)	78.0	0.8775
Split sample	11	0.77(0.72,0.82)	78.6	0.78(0.76,0.81)	92.1	5.03(3.19,7.93)	87.6	0.26(0.15,0.46)	80.9	0.9025
**Models**										
Radiomics model	15	0.73(0.68,0.78)	75.6	0.79(0.77,0.82)	91.1	4.66(3.12,6.97)	82.5	0.36(0.25,0.54)	75.3	0.8749
Combined model	6	0.89(0.81,0.94)	37.8	0.82(0.78,0.86)	78.9	5.06(2.88,8.91)	79.5	0.18(0.09,0.34)	35.4	0.9187

AUC, Area Under Curve; NLR, negative likelihood ratio; PLR, positive likelihood ratio; 2D, Two-Dimensional; 3D, Three-Dimensional.

### Publication bias

**Figure 6:**
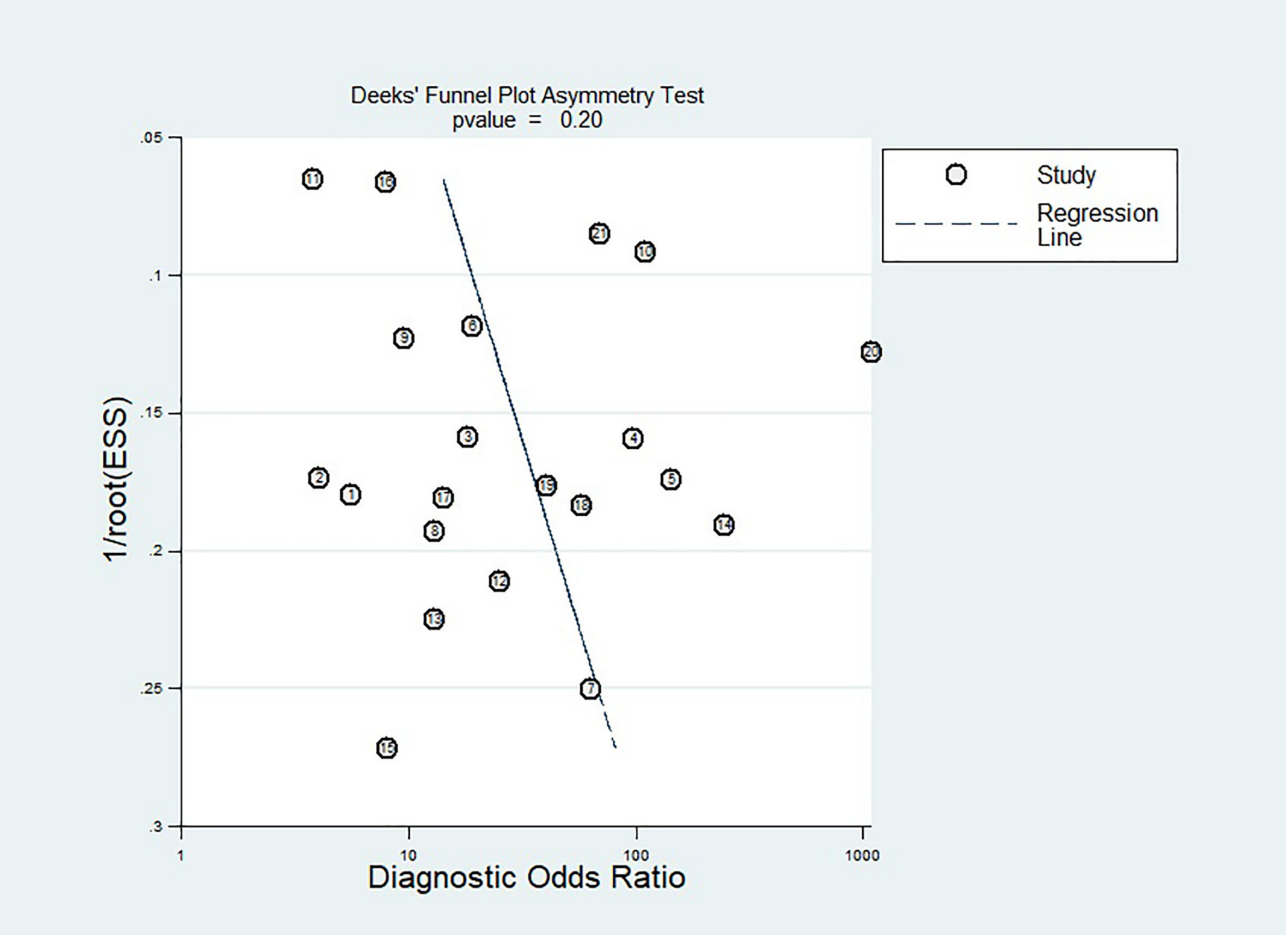
Effective sample size (ESS) funnel plots and the associated regression test of asymmetry, as reported by Deeks et al. A p-value < 0.10 was considered evidence of asymmetry and potential publication bias.

We investigated publication bias for the 21 included papers by first seeing that the funnel plot was symmetric, and then formally assessing it with the Deek test (P=0.20) (**Figure 6**), demonstrating that there was no publication bias.

## Discussion

This systematic review and meta-analysis explored whether radiomics can be accurate in predicting pathological response to neoadjuvant chemoradiotherapy in patients with rectal cancer, using the QUADAS-2 and RQS tools to assess the quality of included studies. The results showed that the radiomic models had high diagnostic value in predicting pCR, with sensitivity, specificity, and AUC of 0.82(95% CI,0.71-0.90), 0.86(95% CI,0.80- 0.91), and 0.91(95% CI,0.88-0.93), respectively. Confirmation of this information will aid in the development of effective therapeutic regimens for rectal cancer patients. For example, If a patient with rectal cancer shows a pCR after neoadjuvant chemoradiotherapy, TME surgery is not required but waiting and observation.

In several studies, first-order features including skewness, kurtosis, entropy, and energy were found to distinguish pathological complete responses from non-pathological complete responses (34, 35, 37, 51–53). Lower kurtosis was found in pCR patients in one study (53), however, this has not been validated in other studies. Second- and higher-order features also have some predictive power. Texture features are changes in image intensity in an image. Texture Analysis (TA) enables researchers to attempt to quantify heterogeneity within the target tumor site, thereby determining the unobservable with more valuable parameters detected (55, 56). Many scholars (35–37, 41, 42, 48, 50–53) have demonstrated that texture features can predict pathological complete response to nCRT. In general, tumors that ultimately failed to achieve pCR after nCRT exhibited elevated or more image heterogeneity, similar to previous findings in breast and lung cancer (57, 58), which demonstrated higher intratumor heterogeneity in patients with a poorer prognosis, including poorer treatment response. Our review also found that combining radiomics signatures across various radiomics categories was more likely to be accurate in predicting nCRT response. This is similar to the literature review by Horvat et al. (59), who found that studies using advanced predictive models had AUCs ranging from 0.72 to 0.93.

The mean RQS score of the 21 included articles was 10.95 (30.4% of the total score). Four items of the RQS in which all included studies performed zero are “Phantom study on all scanners”, “Imaging at multiple time points”, “Cut-off analyses”, and “Cost-effectiveness analysis”. The purpose of a phantom study is to detect different potential feature differences between scanners and suppliers. Many studies included image data from different MRI types (3.0T, 1.5T), vendors (Siemens, Philips), and different medical centers, and phantom studies are a suitable means to gauge these uncertainties and identify features that rely on the vendor. Imaging at multiple time points is based on organ motion or expansion or contraction of the target volume resulting in changes in radiomics characteristics, using remeasurement data (two or more image data sets of a patient acquired in a short period) to obtain stable radiomics features are necessary, especially for the peristaltic hollow organ of the colorectum, however, considering the usual clinical practice work, it is difficult to do this for retrospective studies. Cut-off analyses identified risk groups by medians, previously published cutoff values, or reporting continuous risk variables. Reduce the risk of models with overly optimistic results. Cost-effectiveness analysis is a health economic consideration that argues that cost-quality-adjusted life-year comparisons should be performed with or without radiomics to more accurately determine the economic potential of such studies. The five items where all studies underperformed were “biological correlates”, “Prospective study”, “Potential clinical utility” and “Open science and data”. Only one study combined pathological factors with radiomic features to build predictive models and discussed their biological relevance. Prospective studies are critical enough to link radiomics data to clinical outcomes in appropriate patient populations, however, only two studies were prospective. Three studies considered current and potential applications of models in clinical settings, using decision curves to show the clinical utility of specific models. The openness of data and code contributes to the reproducibility and replicability of radiomics. Radiomics includes multiple complex processes, each one influenced by a variety of factors, including the use of nonstandard nomenclature, the definition of parameters, and the selection of software. If researchers do not reveal these complexities, reproducibility, and replicability in radiomics are impossible. As a result, it is expected that various practical concerns, such as radiomics model repeatability, imaging protocol standardization, model overfitting, and external validation of prediction models, should be thoroughly addressed before transferring these models into routine clinical use.

The QUADAS-2 quality assessment revealed some problems with the 21 studies included in the systematic review. Some studies did not state whether the patients were included in continuous or random sampling, which may lead to selection bias. All studies were at risk of bias on the index test, and it was unclear whether thresholds were used or not pre-specified, which may have led us to overestimate the diagnostic performance of our models. Nine studies did not indicate the time interval between imaging and pathological evaluation of resected tissue after rectal cancer surgery. Future studies should avoid patient selection bias and clarify the time interval between imaging and pathological evaluation of resected tissue after surgery.

Our study was highly heterogeneous, with the heterogeneity of 78.76% and 90.92% for sensitivity and specificity, respectively. We, therefore, performed subgroup analyses using six key factors to explore sources of heterogeneity. In the subgroup analysis, we compared the diagnostic performance of DL and radiomics models, and the diagnostic performance of the DL subgroup was higher than that of the radiomics model, (AUC: 0.97 > 0.85), which may be because DL is trained in the capabilities of multi-layer deep neural networks (60). Compared with ML feature extraction methods, DL is more computationally intensive and can extract more image features (61). ML models are traditionally trained to perform useful tasks using manually specified features retrieved from raw data or features learned by other simple machine learning models (62). DL allows computers to acquire meaningful representations and characteristics automatically, directly from raw data, avoiding this time-consuming and challenging process (63). DL models are dominated by various versions of artificial neural networks, although there are others. The major trait that DL approaches have in common is their emphasis on feature learning: autonomously learning data representations (64). This is the key distinction between DL and more “traditional” ML methodologies. Discovering features and accomplishing a task are combined into one challenge and so improved concurrently during the training phase. However, there are only five DL studies in this meta-analysis. More DL studies are needed to confirm this conclusion. Another subgroup analysis showed that the combined model with clinical factors and radiomics features was more powerful than the radiomics feature alone. Because of the constraints of univariate prediction, its prediction performance is less outstanding, however, the multivariate prediction model can overcome these restrictions. A multifactorial pCR prediction model was established based on this approach, which is also the path for future study, and additional imaging and non-imaging data need to be retrieved to construct stronger prediction models (28).

Two of the twenty-one studies we included used the delta model, which is a new radiomics approach that has been developed that accounts for feature variations at different acquisition times (65). With this method, it is possible to study the impact of changes in characteristics after a specific step in a patient’s workflow (ie, after specific treatment, time, or biological event). Wan and Nardone et al. (41, 48)used delta models to study changes in radiomics parameters throughout the treatment process and showed that the delta model was a good predictor of patient response. Available data suggest that a delta radiomics approach can also successfully predict tumor behavior in terms of synchronous or metachronous distant metastasis (DM), disease-free and overall survival (66, 67).

Our research has several limitations. First, the heterogeneity of research is obvious. We investigated the causes of heterogeneity using subgroup analyses and discovered that heterogeneity was model-related (DL and radiomics), but because heterogeneity was observed in diagnostic test accuracy reviews features (68), we cannot know the source of all the heterogeneity. Second, because the model was not verified, many large-sample studies were excluded from the meta-analysis. Unvalidated models have low relevance, and validation is an essential aspect of a thorough radiomics study (28). Finally, we only evaluated pCR studies and did not include studies on tumor regression grading (TRG) and T downstaging, it is known that pathologic evaluation of TRG and T downstaging is more subjective than pCR evaluation (69, 70). Precise and objective pathological criteria are lacking for TRG and T downstaging.

## Conclusions

In conclusion, our meta-analysis suggests that radiomics is a promising noninvasive approach with a high value for pCR prediction in patients with rectal cancer to neoadjuvant chemoradiotherapy. This has important guiding significance for the individualized treatment of rectal cancer patients in clinical practice. The prediction performance of the DL models for pCR was superior to the radiomics models, and the combined models incorporating clinical factors were superior to the radiomics model alone. Furthermore, more prospective, large-scale, multicenter studies employing radiomics approaches are required in the future to increase pCR preoperative prediction ability.

## Data availability statement

The raw data supporting the conclusions of this article will be made available by the authors, without undue reservation.

## Author contributions

L-LJ designed, developed, and refined the study protocol with contributions from Q-YZ, J-HT, GH, L-PZ, and J-XZ. L-LJ, Q-YZ, and J-HT developed the search strategy and designed the literature search. L-LJ and J-XZ screened titles and abstracts and undertook the data extraction. L-LJ, GH, L-PZ, and J-XZ interpreted the data for the work; L-LJ, Q-YZ drafted the manuscript. All authors were involved in critically revising the draft. All authors contributed to the article and approved the submitted version.

## Conflict of interest

The authors declare that the research was conducted in the absence of any commercial or financial relationships that could be construed as a potential conflict of interest.

## Publisher’s note

All claims expressed in this article are solely those of the authors and do not necessarily represent those of their affiliated organizations, or those of the publisher, the editors and the reviewers. Any product that may be evaluated in this article, or claim that may be made by its manufacturer, is not guaranteed or endorsed by the publisher.
