# Supplementary material for: Artificial intelligence with magnetic resonance imaging for prediction of pathological complete response to neoadjuvant chemoradiotherapy in rectal cancer: A systematic review and meta-analysis
Source: Front Oncol. 2022 Oct 12;12:1026216. doi: 10.3389/fonc.2022.1026216 (PMC9597310; doi:10.3389/fonc.2022.1026216)
Supplement: Supplementary file 1 [file DataSheet_1.docx]

***Supplementary Materials***

Table S1. Literature searching strategies in PubMed, Embase, Cochrane Library and Web of science.

Table S2. Description of the radiomics quality score (RQS) tool.

Table S3. Description of the revised Quality Assessment of Diagnostic Accuracy Studies (QUADAS-2) tool.

Table S4. Methodological quality assessment of each study by RQS tool.

Table S5. Risk of bias and application concerns assessment of each study by the QUADAS-2 tool.

Figure S1. Summary of the risk of bias and applicability assessment.

**Table s1: literature searching strategies in** **PubMed, Embase, Cochrane Library and Web of science.**

| Search | **PubMed Query –June 21, 2022** | Items found |
| --- | --- | --- |
| #19 | #13 AND #17 AND #18 | 441 |
| #18 | "Magnetic Resonance Imaging"[MeSH Terms] OR "MRI Scans"[Title/Abstract] OR "MRI "[Title/Abstract] | 618,139 |
| #17 | #14 OR #15 OR #16 | 286,743 |
| #16 | "Colonic Neoplasms"[MeSH Terms] OR "colonic neoplasms"[Title/Abstract] OR "colonic neoplasm"[Title/Abstract] OR "colon neoplasms"[Title/Abstract] OR "colon neoplasm"[Title/Abstract] OR "cancer of colon"[Title/Abstract] OR "colon cancers"[Title/Abstract] OR "cancer of the colon"[Title/Abstract] OR "colonic cancer"[Title/Abstract] OR "colonic cancers"[Title/Abstract] OR "colon cancer"[Title/Abstract] | 110,360 |
| #15 | "Rectal Neoplasms"[MeSH Terms] OR "rectal neoplasms"[Title/Abstract] OR "rectal neoplasm"[Title/Abstract] OR "rectum neoplasms"[Title/Abstract] OR "rectum neoplasm"[Title/Abstract] OR "rectal tumors"[Title/Abstract] OR "rectal tumor"[Title/Abstract] OR "cancer of rectum"[Title/Abstract] OR "rectum cancers"[Title/Abstract] OR "rectal cancer"[Title/Abstract] OR "rectal cancers"[Title/Abstract] OR "rectum cancer"[Title/Abstract] OR "cancer of the rectum"[Title/Abstract] | 61,207 |
| #14 | "Colorectal Neoplasms"[MeSH Terms] OR "colorectal neoplasms"[Title/Abstract] OR "colorectal neoplasm"[Title/Abstract] OR "colorectal tumors"[Title/Abstract] OR "colorectal tumor"[Title/Abstract] OR "colorectal cancer"[Title/Abstract] OR "colorectal cancers"[Title/Abstract] OR "colorectal carcinoma"[Title/Abstract] OR "colorectal carcinomas"[Title/Abstract] | 265,359 |
| #13 | #6 OR #12 | 850,499 |
| #12 | #7 OR #8 OR #9 OR #10 OR #11 | 655,655 |
| #11 | "random forest"[Title/Abstract] OR "random forest classifier"[Title/Abstract] OR "RFC"[Title/Abstract] OR "RF"[Title/Abstract] OR "CNN"[Title/Abstract] OR "resnet model"[Title/Abstract] OR "gradient boosting machine"[Title/Abstract] OR "GBM"[Title/Abstract] OR "NNet"[Title/Abstract] OR "latent dirichlet allocation"[Title/Abstract] OR "LDA"[Title/Abstract] OR "texture"[Title/Abstract] OR "LASSO"[Title/Abstract] OR "boosting"[Title/Abstract] OR "radiomics"[Title/Abstract] OR "radiogenomic*"[Title/Abstract] | 156,423 |
| #10 | "computer neural network"[Title/Abstract] OR "computer neural networks"[Title/Abstract] OR "neural network model"[Title/Abstract] OR "neural network models"[Title/Abstract] OR "computational neural networks"[Title/Abstract] OR "computational neural network"[Title/Abstract] OR "Perceptrons"[Title/Abstract] OR "Perceptron"[Title/Abstract] OR "connectionist models"[Title/Abstract] OR "connectionist model"[Title/Abstract] | 9,565 |
| #9 | "Logistic Models"[MeSH Terms] OR "logistic models"[Title/Abstract] OR "logistic model"[Title/Abstract] OR "logit models"[Title/Abstract] OR "logit model"[Title/Abstract] OR "logistic regression"[Title/Abstract] OR "logistic regressions"[Title/Abstract] | 437,623 |
| #8 | "Decision Trees"[MeSH Terms] OR "decision tree"[Title/Abstract] OR "decision trees"[Title/Abstract] OR "DT"[Title/Abstract] | 48,345 |
| #7 | "Support Vector Machine"[MeSH Terms] OR "support vector machine"[Title/Abstract] OR "support vector machines"[Title/Abstract] OR "support vector network"[Title/Abstract] OR "support vector networks"[Title/Abstract] | 23,819 |
| #6 | #1 OR #2 OR #3 OR #4 OR #5 | 243,679 |
| #5 | "Unsupervised Machine Learning"[MeSH Terms] | 702 |
| #4 | "Supervised Machine Learning"[MeSH Terms] OR "supervised machine learning"[Title/Abstract] OR "semi supervised learning"[Title/Abstract] OR "semi supervised learning"[Title/Abstract] OR "inductive machine learning"[Title/Abstract] OR "active machine learning"[Title/Abstract] OR "learning from labeled data"[Title/Abstract] | 12,852 |
| #3 | "Deep Learning"[MeSH Terms] OR "deep learning"[Title/Abstract] OR "hierarchical learning"[Title/Abstract] | 34,563 |
| #2 | "Machine Learning"[MeSH Terms] OR "machine learning"[Title/Abstract] OR "transfer learning"[Title/Abstract] | 89,784 |
| #1 | "Artificial Intelligence"[MeSH Terms] OR "artificial intelligence"[Title/Abstract] OR "computational intelligence"[Title/Abstract] OR "machine intelligence"[Title/Abstract] OR "computer reasoning"[Title/Abstract] OR "AI"[Title/Abstract] OR "computer vision systems"[Title/Abstract] OR "computer vision system"[Title/Abstract] OR "knowledge acquisition"[Title/Abstract] OR "knowledge representation"[Title/Abstract] OR "knowledge representations"[Title/Abstract] | 199,816 |

| Search | **Embase.com Query –June 21, 2022** | Items found |
| --- | --- | --- |
| #18 | #16 AND #17 | 491 |
| #17 | 'Magnetic Resonance Imaging ':ab,ti OR 'MRI Scans':ab,ti OR 'MRI':ab,ti | 657,080 |
| #16 | #11 AND #15 | 15,071 |
| #15 | #12 OR #13 OR #14 | 375,594 |
| #14 | 'colon cancer'/exp OR 'colon cancer':ab,ti OR 'colonic cancer':ab,ti | 146,421 |
| #13 | 'rectum cancer'/exp OR 'cancer rectum':ab,ti OR 'rectal cancer':ab,ti OR 'rectal malignancy':ab,ti OR 'rectum malignancy':ab,ti | 64,130 |
| #12 | 'colorectal cancer'/exp | 351,861 |
| #11 | #1 OR #2 OR #3 OR #4 OR #5 OR #6 OR #7 OR #8 OR #9 OR #10 | 1,111,714 |
| #10 | 'support vector machine'/exp OR 'support vector machine':ab,ti OR 'support vector':ab,ti OR 'support vector classification':ab,ti OR 'support vector classifier':ab,ti OR 'support vector classifiers':ab,ti OR 'support vector machines':ab,ti OR 'support vector network':ab,ti OR 'support vector regression':ab,ti OR 'support vectors':ab,ti OR svc:ab,ti OR svm:ab,ti OR svr:ab,ti | 63,875 |
| #9 | 'resnet model':ab,ti OR 'gradient boosting machine':ab,ti OR nnet:ab,ti OR 'latent dirichlet allocation':ab,ti OR texture:ab,ti OR lasso:ab,ti OR radiogenomic* OR 'radiomics'/exp OR radiomics | 58,964 |
| #8 | 'radiomics'/exp | 5,182 |
| #7 | 'random forest'/exp OR 'random forest':ab,ti OR 'random decision forest':ab,ti OR 'random forest algorithm':ab,ti OR 'random forests':ab,ti | 22,999 |
| #6 | 'artificial neural network'/exp OR 'artificial neural network':ab,ti OR 'algorithmic neural network':ab,ti OR 'ann approach':ab,ti OR 'ann method':ab,ti OR 'ann methodology':ab,ti OR 'ann methods':ab,ti OR 'ann model':ab,ti OR 'ann modeling':ab,ti OR 'ann modelling':ab,ti OR 'ann models':ab,ti OR 'ann output':ab,ti OR 'ann technique':ab,ti OR 'ann techniques':ab,ti OR 'ann training':ab,ti OR anns:ab,ti OR 'artificial neural networks':ab,ti OR 'artificial nns':ab,ti OR 'computational neural network':ab,ti OR 'computer neural network':ab,ti OR 'computerized neural network':ab,ti OR 'connectionist model':ab,ti OR 'connectionist network':ab,ti OR 'connectionist neural network':ab,ti OR 'connectionist system':ab,ti OR 'mathematical neural network':ab,ti OR 'neural network artificial':ab,ti OR 'neural network algorithm':ab,ti OR 'neural network model':ab,ti | 76,591 |
| #5 | 'decision tree'/exp OR 'decision trees':ab,ti OR 'decision tree':ab,ti | 23,503 |
| #4 | 'statistical model'/exp OR 'likelihood functions':ab,ti OR 'linear model':ab,ti OR 'linear models':ab,ti OR 'logistic models':ab,ti OR 'statistic model':ab,ti OR 'statistical models':ab,ti OR 'statistics model':ab,ti | 684,047 |
| #3 | 'deep learning'/exp OR 'hierarchical learning':ab,ti OR 'deep learning':ab,ti | 38,149 |
| #2 | 'machine learning'/exp OR 'machine learning':ab,ti OR 'learning machine':ab,ti OR 'learning machines':ab,ti | 329,566 |
| #1 | 'artificial intelligence'/exp OR 'artificial intelligence':ab,ti OR 'machine intelligence':ab,ti | 81,337 |

| Search | **The Cochrane Library Query –June 21, 2022** | Items found |
| --- | --- | --- |
| #25 | #13 AND #23 AND #24 | 19 |
| #24 | (Magnetic Resonance Imaging):ti,ab,kw OR (MRI Scans):ti,ab,kw OR (MRI):ti,ab,kw | 38,784 |
| #23 | #16 OR #19 OR #22 | 26,464 |
| #22 | #20 OR #21 | 8,053 |
| #21 | (Colonic Neoplasms):ti,ab,kw OR (Colonic Neoplasm):ti,ab,kw OR (Colon Neoplasms):ti,ab,kw OR (Colon Neoplasm):ti,ab,kw OR (Cancer of Colon):ti,ab,kw OR(Colon Cancers):ti,ab,kw OR (Cancer of the Colon):ti,ab,kw OR (Colonic Cancer):ti,ab,kw OR (Colonic Cancers):ti,ab,kw OR (Colon Cancer):ti,ab,kw | 8,026 |
| #20 | MeSH descriptor: [Colonic Neoplasms] explode all trees | 1,913 |
| #19 | #17 OR #18 | 7,939 |
| #18 | (Rectal Neoplasms):ti,ab,kw OR (Rectal Neoplasm):ti,ab,kw OR (Rectum Neoplasms):ti,ab,kw OR (Rectum Neoplasm):ti,ab,kw OR (Rectal Tumors):ti,ab,kw OR (Rectal Tumor)ti,ab,kw OR (Cancer of Rectum):ti,ab,kw OR (Rectum Cancers):ti,ab,kw OR (Rectal Cancer):ti,ab,kw OR (Rectal Cancers):ti,ab,kw OR (Rectum Cancer):ti,ab,kw OR (Cancer of the Rectum):ti,ab,kw | 7,825 |
| #17 | MeSH descriptor: [Rectal Neoplasms] explode all trees | 2,025 |
| #16 | #14 OR #15 | 20,382 |
| #15 | (Colorectal Neoplasms):ti,ab,kw OR (Colorectal Neoplasm):ti,ab,kw OR (Colorectal Tumors):ti,ab,kw OR (Colorectal Tumor):ti,ab,kw OR(Colorectal Cancer):ti,ab,kw OR (Colorectal Cancers):ti,ab,kw OR (Colorectal Carcinoma):ti,ab,kw OR (Colorectal Carcinomas):ti,ab,kw | 17,954 |
| #14 | MeSH descriptor: [Colorectal Neoplasms] explode all trees | 9,249 |
| #13 | #3 OR #6 OR #9 OR #12 | 6254 |
| #12 | #10 OR #11 | 232 |
| #11 | (Semi-supervised Learning):ti,ab,kw OR (Semi supervised Learning):ti,ab,kw OR (Inductive Machine Learning):ti,ab,kw OR (Active Machine Learning):ti,ab,kw OR (Learning from Labeled Data):ti,ab,kw | 210 |
| #10 | MeSH descriptor: [Supervised Machine Learning] explode all trees | 25 |
| #9 | #7 OR #8 | 1,289 |
| #8 | (Deep Learning):ti,ab,kw OR (Hierarchical Learning):ti,ab,kw | 1,289 |
| #7 | MeSH descriptor: [Deep Learning] explode all trees | 65 |
| #6 | #4 OR #5 | 3,378 |
| #5 | (Machine Learning):ti,ab,kw OR (Transfer Learning):ti,ab,kw | 3,307 |
| #4 | MeSH descriptor: [Machine Learning] explode all trees | 252 |
| #3 | #1 OR #2 | 2,510 |
| #2 | (Computational Intelligence):ti,ab,kw OR (Artificial Intelligence):ti,ab,kw OR (Computational Intelligence):ti,ab,kw OR (Machine Intelligence):ti,ab,kw OR (Computer Reasoning):ti,ab,kw | 1,327 |
| #1 | MeSH descriptor: [Artificial Intelligence] explode all trees | 1,457 |

| Search | **Web Query –June 21, 2022** | Items found |
| --- | --- | --- |
| #4 | #3 AND #2 AND #1 | 611 |
| #3 | TS=( Magnetic Resonance Imaging OR MRI Scans OR MRI) | 570,758 |
| #2 | TS= (colorectal neoplasms OR colorectal tumors OR colorectal cancer OR colorectal carcinomas OR rectal neoplasm OR rectum neoplasm OR rectal tumor OR cancer of rectum OR colonic neoplasms OR colon neoplasm OR colon cancers OR colonic cancer) | 345,469 |
| #1 | TS=(artificial intelligence OR computational intelligence OR machine intelligence OR Machine Learning OR transfer learning OR deep learning OR hierarchical learning OR supervised machine learning OR semi supervised learning OR inductive machine learning OR Unsupervised Machine Learning OR support vector machine OR support vector network OR decision tree OR logistic models OR computer neural network OR neural network model OR connectionist models OR random forest OR random forest classifier OR RFC OR RF OR resnet model OR gradient boosting machine OR latent dirichlet allocation OR texture OR LASSO OR boosting OR radiomics OR radiogenomic*) | 1,732,726 |

**Table s2: Description of the radiomics quality score (RQS) tool.**

|  | **Criteria** | **Points** |
| --- | --- | --- |
| **1** | **Image protocol quality** - well-documented image protocols (for example, contrast, slice thickness, energy, etc.) and/or usage of public image protocols allowreproducibility/replicability | + 1 (if protocols are well-documented)  +1 (if public protocol is used) |
| **2** | **Multiple segmentations** - possible actions are: segmentation by different physicians/algorithms/software, perturbing segmentations by (random) noise, segmentation at different breathing cycles. Analyse feature robustness to segmentation variabilities | + 1 |
| **3** | **Phantom study on all scanners** - detect inter-scanner differences and vendor-dependent features. Analyse feature robustness to these sources of variability | + 1 |
| **4** | **Imaging at multiple time points** - collect images of individuals at additional time points. Analyse feature robustness to temporal variabilities (for example, organ movement, organ expansion/ shrinkage) | + 1 |
| **5** | **Feature reduction** or adjustment for multiple testing - decreases the risk of overfitting. Overfitting is inevitable if the number of features exceeds the number of samples. Consider feature robustness when selecting features | - 3 (if neither measure is implemented)  +3 (if either measure is implemented) |
| **6** | **Multivariable analysis** with non radiomics features (for example, EGFR mutation) - is expected to provide a more holistic model. Permits correlating/inferencing between radiomics and non radiomics features | + 1 |
| **7** | Detect and discuss **biological correlates** - demonstration of phenotypic differences (possibly associated with underlying gene–protein expression patterns) deepens understanding of radiomics and biology | + 1 |
| **8** | **Cut-off analyses** - determine risk groups by either the median, a previously published cut-off or report a continuous risk variable. Reduces the risk of reporting overly optimistic results | + 1 |
| **9** | **Discrimination statistics** - report discrimination statistics (for example, C-statistic, ROC curve, AUC) and their statistical significance (for example, p-values, confidence intervals). One can also apply resampling method (for example, bootstrapping, cross-validation) | + 1 (if a discrimination statistic and its statistical significance are reported)  +1 (if a resampling method technique is also applied) |
| **10** | **Calibration statistics** - report calibration statistics (for example, Calibration-in-the-large/slope, calibration plots) and their statistical significance (for example, *P*-values, confidence intervals). One can also apply resampling method (for example, bootstrapping, cross-validation) | + 1 (if a calibration statistic and its statistical significance are reported)  +1 (if a resampling method technique is also applied) |
| **11** | **Prospective study** registered in a trial database - provides the highest level of evidence supporting the clinical validity and usefulness of the radiomics biomarker | + 7 (for prospective validation of a radiomics signature in an appropriate trial) |
| **12** | **Validation** - the validation is performed without retraining and without adaptation of the cut-off value, provides crucial information with regard to credible clinical performance | - 5 (if validation is missing)  +2 (if validation is based on a dataset  from the same institute)  +3 (if validation is based on a dataset from another institute)  +4 (if validation is based on two datasets from two distinct institutes)  +4 (if the study validates a previously published signature)  +5 (if validation is based on three or more datasets from distinct institutes)  *Datasets should be of comparable size and should have at least  10 events per model feature |
| **13** | **Comparison to ‘gold standard’** - assess the extent to which the model agrees with/is superior to the current ‘gold standard’ method (for example, TNM-staging for survival prediction). This comparison shows the added value of radiomics | +2 |
| **14** | **Potential clinical utility** - report on the current and potential application of the model in a clinical setting (for example, decision curve analysis). | +2 |
| **15** | **Cost-effectiveness analysis** - report on the cost-effectiveness of the clinical application (for example, QALYs generated) | +1 |
| **16** | **Open science and data** - make code and data publicly available. Open science facilitates knowledge transfer and reproducibility of the study | + 1 (if scans are open source)  + 1 (if region of interest  segmentations are open source)  + 1 (if code is open source)  + 1 (if radiomics features are calculated on a set of representative ROIs and the calculated features andrepresentative ROIs are open source) |
|  | Total points (36=100%) |  |

**Table s3: Description of the revised Quality Assessment of Diagnostic Accuracy Studies (QUADAS-2) tool**

| **Domain** | **Patient selection** | **Index test** | **Reference**  **standard** | **Flow and timing** |
| --- | --- | --- | --- | --- |
| **Signalling**  **questions**  **(yes, no, or**  **unclear)** | Was a consecutive or random sample of patients enrolled?  Was a case-control design avoided?  Did the study avoid inappropriate  exclusions? | Were the index test results interpreted  without knowledge of the results of the reference standard?  If a threshold was used, was it prespecified? | Is the the reference standard likely to correctly classify  the target condition?  Were the reference standard results interpreted without  knowledge of the results of the index test? | Was there an appropriate interval between index test  and reference standard?  Did all patients receive a reference standard?  Did all patients receive the same reference standard?  Were all patients included in the analysis? |
| **Risk of bias**  **(high, low,**  **or unclear)** | Could the selection of patients have  introduced bias? | Could the conduct or interpretation of the index test have introduced bias? | Could the reference standard, its conduct, or its interpretation have introduced bias? | Could the patient flow have introduced bias? |
| **Concerns**  **about**  **applicability**  **(high, low,**  **or unclear)** | Are there concerns that the included patients do not match the review question? | Are there concerns that the index test, its conduct, or its interpretation differ from the review question? | Are there concerns that the target condition as defined by the reference standard does not match the review question? | - |

Source: Whiting PF, Rutjes AW, Westwood ME, et al; QUADAS-2 Group. QUADAS-2: a revised tool for the quality assessment of diagnostic accuracy studies. Ann Intern Med. 2011 Oct 18;155(8):529-36. doi: 10.7326/0003-4819-155-8-201110180-00009.

**Table s4. Methodological quality assessment of each study by the RQS tool**

| **Study ID** | **Image**  **protocol**  **quality** | **Multiple**  **segmenta**  **tions** | **Phanto**  **m study** | **Imagin**  **g at**  **multipl**  **e time**  **points** | **Feature**  **reductio**  **n** | **Multivariabl**  **e analysis**  **with non**  **radiomics**  **features** | **Biologica**  **l**  **correlate**  **s** | **Cut-off**  **analyse**  **s** | **Discriminatio**  **n statistics** | **Calibratio**  **n statistics** | **Prospectiv**  **e study** | **Validatio**  **n** | **Compariso**  **n to ‘gold**  **standard** | **Potentia**  **l clinical**  **utility** | **Cost-**  **effectivenes**  **s analysis** | **Open**  **scienc**  **e and**  **data** | **Total**  **point**  **s** |
| --- | --- | --- | --- | --- | --- | --- | --- | --- | --- | --- | --- | --- | --- | --- | --- | --- | --- |
| **Antunes 2021** | 1 | 1 | 0 | 0 | 3 | 1 | 0 | 0 | 2 | 0 | 0 | 4 | 0 | 0 | 0 | 0 | 12  (33%) |
| **Boldrini 2022** | 0 | 1 | 0 | 0 | 3 | 0 | 0 | 0 | 1 | 0 | 0 | 3 | 0 | 0 | 0 | 0 | 8  (22%) |
| **Bulens 2021** | 1 | 1 | 0 | 0 | 3 | 0 | 0 | 0 | 2 | 2 | 0 | 3 | 0 | 0 | 0 | 1 | 13  (36%) |
| **Cheng 2021** | 1 | 1 | 0 | 0 | 3 | 1 | 0 | 0 | 1 | 1 | 0 | 2 | 2 | 2 | 0 | 0 | 14  (39%) |
| **Cui 2019** | 1 | 1 | 0 | 0 | 3 | 1 | 0 | 0 | 2 | 2 | 0 | 2 | 0 | 2 | 0 | 0 | 14  (39%) |
| **Feng 2022** | 1 | 1 | 0 | 0 | 3 | 1 | 1 | 0 | 2 | 0 | 7 | 5 | 0 | 2 | 0 | 1 | 24  (67%) |
| **Horvat 2018** | 1 | 0 | 0 | 0 | 0 | 1 | 0 | 0 | 1 | 0 | 0 | 2 | 2 | 0 | 0 | 0 | 7  (19%) |
| **Horvat 2022** | 1 | 1 | 0 | 0 | 3 | 0 | 0 | 0 | 2 | 0 | 0 | 3 | 0 | 0 | 0 | 0 | 10  (28%) |
| **Jang 2021** | 1 | 0 | 0 | 0 | 0 | 0 | 0 | 0 | 1 | 0 | 0 | 2 | 0 | 0 | 0 | 0 | 4  (11%) |
| **Jin 2021** | 1 | 0 | 0 | 0 | 0 | 0 | 0 | 0 | 2 | 0 | 0 | 4 | 0 | 0 | 0 | 1 | 8  (22%) |
| **Lee 2021** | 1 | 1 | 0 | 0 | 3 | 0 | 0 | 0 | 1 | 0 | 0 | 2 | 0 | 0 | 0 | 0 | 7  (19%) |
| **Nardone 2022** | 1 | 1 | 0 | 0 | 0 | 0 | 0 | 0 | 1 | 0 | 0 | 4 | 2 | 0 | 0 | 0 | 9  (25%） |
| **Pang 2021** | 1 | 1 | 0 | 0 | 0 | 0 | 0 | 0 | 1 | 0 | 0 | 3 | 0 | 0 | 0 | 0 | 6  (17%) |
| **Rengo 2022** | 1 | 1 | 0 | 0 | 3 | 0 | 0 | 0 | 2 | 0 | 0 | 3 | 2 | 0 | 0 | 0 | 12  (33%) |
| **Shaish 2020** | 1 | 0 | 0 | 0 | 3 | 0 | 0 | 0 | 2 | 0 | 0 | 3 | 0 | 0 | 0 | 0 | 9  (25%) |
| **Shin 2022** | 1 | 1 | 0 | 0 | 3 | 0 | 0 | 0 | 2 | 0 | 0 | 2 | 0 | 0 | 0 | 1 | 10  (28%) |
| **Wan 2019** | 1 | 1 | 0 | 0 | 3 | 1 | 0 | 0 | 2 | 0 | 0 | 2 | 0 | 0 | 0 | 0 | 10  (28%) |
| **Wan 2020** | 1 | 1 | 0 | 0 | 3 | 0 | 0 | 0 | 2 | 0 | 0 | 2 | 2 | 0 | 0 | 0 | 11  (31%) |
| **Yi 2019** | 1 | 1 | 0 | 0 | 3 | 1 | 0 | 0 | 1 | 0 | 0 | 2 | 0 | 0 | 0 | 0 | 9  (25%) |
| **Zhang 2020** | 1 | 1 | 0 | 0 | 3 | 0 | 0 | 0 | 2 | 2 | 7 | 2 | 2 | 2 | 0 | 0 | 22  (61%) |
| **Zhu 2022** | 1 | 1 | 0 | 0 | 3 | 0 | 0 | 0 | 2 | 1 | 0 | 2 | 0 | 0 | 0 | 1 | 11  (31%) |

**Table s5. Risk of bias and application concerns assessment of each study by the QUADAS-2 tool**

|  | Risk of Bias |  |  |  | Applicability Concerns | | |
| --- | --- | --- | --- | --- | --- | --- | --- |
| Study ID | Patient  selection | Index test | Reference  standard | Flow and timing | Patient  selection | Index  test | Reference  standard |
| Antunes 2021 | Unclear | High | Low | Low | Low | High | Low |
| Boldrini 2022 | Low | High | Low | Unclear | Low | High | Low |
| Bulens 2021 | Low | Unclear | Low | Low | Low | Low | Low |
| Cheng 2021 | Low | Unclear | Low | Unclear | Low | Low | Low |
| Cui 2019 | Low | Unclear | Low | Unclear | Low | Low | Low |
| Feng 2022 | Unclear | Unclear | Low | Low | Low | Low | Low |
| Horvat 2018 | Low | Unclear | Low | Low | Low | Low | Low |
| Horvat 2022 | Low | Unclear | Low | High | Low | Low | Low |
| Jang 2021 | Unclear | High | Low | Low | Low | High | Low |
| Jin 2021 | Unclear | High | Low | Low | Low | High | Low |
| Lee 2021 | High | Unclear | Unclear | High | High | Low | Unclear |
| Nardone 2022 | Unclear | High | Low | Unclear | Low | High | Low |
| Pang 2021 | Unclear | Unclear | Low | Unclear | Low | Low | Low |
| Rengo 2022 | Unclear | Unclear | Low | Low | Low | Low | Low |
| Shaish 2020 | Unclear | High | Low | Unclear | Low | High | Low |
| Shin 2022 | Low | Unclear | Low | Low | Low | Low | Low |
| Wan 2019 | Low | Unclear | Low | Low | Low | Low | Low |
| Wan 2020 | Low | Unclear | Low | Low | Low | Low | Low |
| Yi 2019 | Unclear | Unclear | Low | Low | Low | Low | Low |
| Zhang 2020 | Low | Unclear | Low | Low | Low | Low | Low |
| Zhu 2022 | Unclear | Unclear | Low | Unclear | Low | Low | Low |


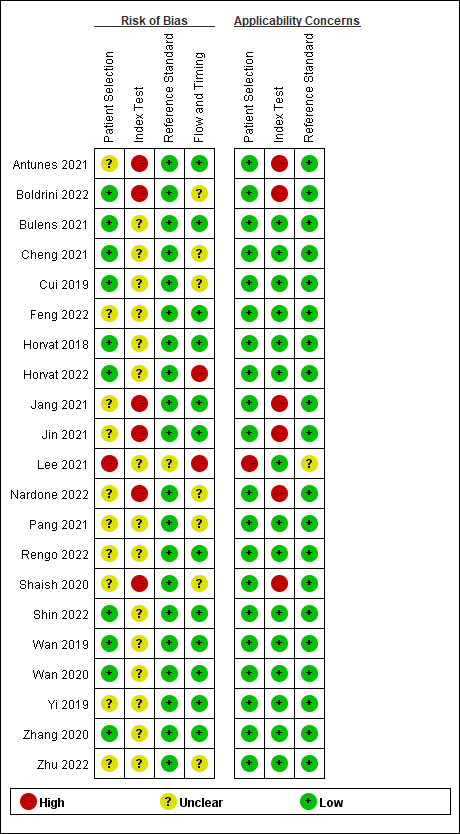


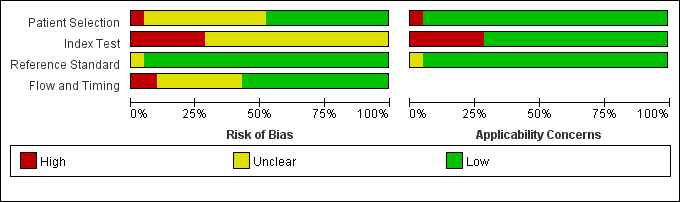


**Figure s1.** Summary of the risk of bias and applicability assessment: authors’ judgement for each domain of each included study was reviewed. The proportion of included studies that indicated low, unclear, or high risk and applicability concerns are shown in green, yellow, and red, respectivel.
